# Recoloring tomato fruit by CRISPR/Cas9-mediated multiplex gene editing

**DOI:** 10.1093/hr/uhac214

**Published:** 2022-09-19

**Authors:** Tianxia Yang, Muhammad Ali, Lihao Lin, Ping Li, Hongju He, Qiang Zhu, Chuanlong Sun, Ning Wu, Xiaofei Zhang, Tingting Huang, Chang-Bao Li, Chuanyou Li, Lei Deng

**Affiliations:** State Key Laboratory of Plant Genomics, National Center for Plant Gene Research (Beijing), Institute of Genetics and Developmental Biology, Innovation Academy for Seed Design, Chinese Academy of Sciences, Beijing 100101, China; CAS Center for Excellence in Biotic Interactions, University of Chinese Academy of Sciences, Beijing 100049, China; State Key Laboratory of Plant Genomics, National Center for Plant Gene Research (Beijing), Institute of Genetics and Developmental Biology, Innovation Academy for Seed Design, Chinese Academy of Sciences, Beijing 100101, China; CAS Center for Excellence in Biotic Interactions, University of Chinese Academy of Sciences, Beijing 100049, China; State Key Laboratory of Plant Genomics, National Center for Plant Gene Research (Beijing), Institute of Genetics and Developmental Biology, Innovation Academy for Seed Design, Chinese Academy of Sciences, Beijing 100101, China; CAS Center for Excellence in Biotic Interactions, University of Chinese Academy of Sciences, Beijing 100049, China; Institute of Vegetable, Qingdao Academy of Agricultural Sciences, Qingdao, Shandong 266100, China; Institute of Agri-food Processing and Nutrition, Beijing Academy of Agriculture and Forestry Sciences, Beijing 100097, China; State Key Laboratory of Plant Genomics, National Center for Plant Gene Research (Beijing), Institute of Genetics and Developmental Biology, Innovation Academy for Seed Design, Chinese Academy of Sciences, Beijing 100101, China; CAS Center for Excellence in Biotic Interactions, University of Chinese Academy of Sciences, Beijing 100049, China; State Key Laboratory of Plant Genomics, National Center for Plant Gene Research (Beijing), Institute of Genetics and Developmental Biology, Innovation Academy for Seed Design, Chinese Academy of Sciences, Beijing 100101, China; CAS Center for Excellence in Biotic Interactions, University of Chinese Academy of Sciences, Beijing 100049, China; State Key Laboratory of Plant Genomics, National Center for Plant Gene Research (Beijing), Institute of Genetics and Developmental Biology, Innovation Academy for Seed Design, Chinese Academy of Sciences, Beijing 100101, China; CAS Center for Excellence in Biotic Interactions, University of Chinese Academy of Sciences, Beijing 100049, China; State Key Laboratory of Plant Genomics, National Center for Plant Gene Research (Beijing), Institute of Genetics and Developmental Biology, Innovation Academy for Seed Design, Chinese Academy of Sciences, Beijing 100101, China; CAS Center for Excellence in Biotic Interactions, University of Chinese Academy of Sciences, Beijing 100049, China; Institute of Vegetable, Qingdao Academy of Agricultural Sciences, Qingdao, Shandong 266100, China; Key Laboratory of Biology and Genetic Improvement of Horticultural Crops (North China), Ministry of Agriculture, Beijing Vegetable Research Center, Beijing Academy of Agriculture and Forestry Sciences, Beijing 100097, China; State Key Laboratory of Plant Genomics, National Center for Plant Gene Research (Beijing), Institute of Genetics and Developmental Biology, Innovation Academy for Seed Design, Chinese Academy of Sciences, Beijing 100101, China; CAS Center for Excellence in Biotic Interactions, University of Chinese Academy of Sciences, Beijing 100049, China; State Key Laboratory of Plant Genomics, National Center for Plant Gene Research (Beijing), Institute of Genetics and Developmental Biology, Innovation Academy for Seed Design, Chinese Academy of Sciences, Beijing 100101, China; CAS Center for Excellence in Biotic Interactions, University of Chinese Academy of Sciences, Beijing 100049, China

## Abstract

Fruit color is an important horticultural trait, which greatly affects consumer preferences. In tomato, fruit color is determined by the accumulation of different pigments, such as carotenoids in the pericarp and flavonoids in the peel, along with the degradation of chlorophyll during fruit ripening. Since fruit color is a multigenic trait, it takes years to introgress all color-related genes in a single genetic background via traditional crossbreeding, and the avoidance of linkage drag during this process is difficult. Here, we proposed a rapid breeding strategy to generate tomato lines with different colored fruits from red-fruited materials by CRISPR/Cas9-mediated multiplex gene editing of three fruit color-related genes (*PSY1*, *MYB12*, and *SGR1*). Using this strategy, the red-fruited cultivar ‘Ailsa Craig’ has been engineered to a series of tomato genotypes with different fruit colors, including yellow, brown, pink, light-yellow, pink-brown, yellow-green, and light green. Compared with traditional crossbreeding, this strategy requires less time and can obtain transgene-free plants with different colored fruits in less than 1 year. Most importantly, it does not alter other important agronomic traits, like yield and fruit quality. Our strategy has great practical potential for tomato breeding and serves as a reference for improving multigene-controlled traits of horticultural crops.

## Introduction

Tomato (*Solanum lycopersicum*) is one of the most consumed vegetables worldwide and provides a classical model system for studying fruit biology. Fruit color is an important horticultural trait of tomato and often affects the purchasing decision of consumers [1]. The color of tomato fruit is determined by the pigments contained in its peel and pericarp. Some of the pigments are known to prevent cardiovascular disease and reduce obesity in humans [2, 3].

Tomato fruits display a wide range of colors, such as red, orange, pink, yellow, brown, green, purple, and even white, which are determined by the levels and ratios of different pigments. The red color of ripe tomato fruit is mainly caused by the accumulation of all-*trans*-lycopene (a carotenoid) and naringenin chalcone (NarCh) as well as by the degradation of chlorophyll occurring during fruit ripening [4]. Mutation of carotenoid biosynthesis genes altered carotene composition, thereby resulting in different fruit colors. While the loss-of-function of locus *r* gene *Phytoene Synthase 1* (*PSY1*) led to yellow fruit [5], *tangerine* (locus *t*) and *fruit carotenoid-deficient* mutants produced orange fresh fruits because of mutations in the *CRTISO* and *IDI1* genes, respectively [6–8]. Additionally, mutations in the *CrtL-b* and *CrtL-e* genes, which encode lycopene *β*-cyclase and *ε*-cyclase, respectively, caused the development of orange color in ripe fruit because of the accumulation of carotene at the expense of lycopene [9]. Besides carotenoids, flavonoids also play a significant role in determining the color of tomato fruit [10]. The peel of tomato fruit is the predominant source of flavonoid, since flavonoid biosynthesis genes are not expressed in the flesh [11, 12]. Tomato fruit with red-colored flesh exhibited yellow-colored peel because of the accumulation of the yellow-colored flavonoid NarCh in the peel. Genetic studies revealed that pink tomato fruit color is a monogenic trait controlled by the recessive *yellow* (*y*) locus [10]. The *Y* gene encodes an R2R3-MYB transcription factor (*MYB12*), which plays a critical function in regulating the production of NarCh in the tomato fruit. Knockout mutation of *MYB12* disrupted NarCh accumulation, resulting in colorless peel, which ultimately led to the production of pink-colored fruit [13, 14]. In general, a sharp decline in chlorophyll content and a concomitant increase in the carotenoid content occur during tomato fruit ripening. However, mutation in *STAY-GREEN 1* (*SGR1*) inhibits chlorophyll degradation during ripening, which, combined with the accumulation of lycopene during ripening, leads to the production of brown-colored fruit [15]. Overall, the color of tomato fruit is determined by the accumulation of carotenoids and flavonoids, as well as the degradation of chlorophyll. Thus, a wide range of fruit colors can be obtained by manipulating the ratios of these three pigments.

Different colored fruits can be obtained by traditional crossbreeding. However, this approach is laborious and time-consuming, and often causes problems like linkage drag [16]. The CRISPR/Cas9 (clustered regularly interspaced short palindromic repeats/CRISPR-associated 9) system is a powerful, rapid, and precise breeding technique that has been used in many crop plants, such as rice, wheat, maize, soybean, and tomato [14, 16–20], for controlling complex and polygenic traits. Moreover, CRISPR-edited crops are not under the same stringent regulations as the traditionally generated genetically modified crops. Thus, it is believed that CRISPR technology will revolutionize the pace of crop breeding in the future.

**Figure 1:**
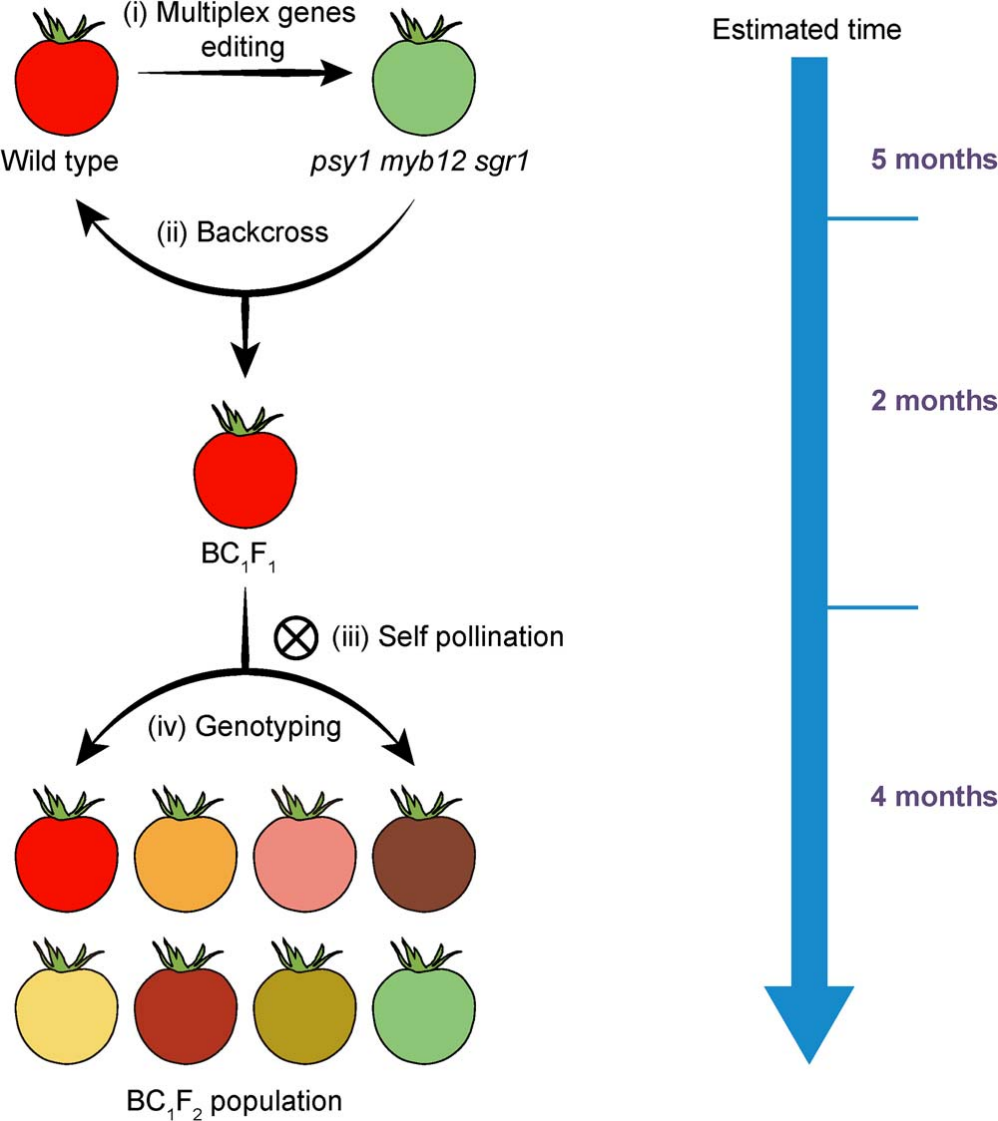
Schematic diagram showing the generation of tomato lines producing different colored fruits. The green-fruited *psy1 myb12 sgr1* triple mutant was generated using the CRISPR/Cas9 system and backcrossed with the red-fruited WT cultivar ‘Ailsa Craig’.

Here, we developed a rapid breeding strategy to generate tomato lines with different colored fruits from red-fruited materials by CRISPR/Cas9-mediated multiplex gene editing (Fig. 1). First, we created a green-fruited tomato genotype from the red-fruited cultivar ‘Ailsa Craig’ through simultaneous knockout mutations of *PSY1*, *MYB12*, and *SGR1*. Then, we backcrossed the *psy1 myb12 sgr1* triple mutant in the T_0_ generation with wild-type (WT) plants to generate BC_1_F_1_ hybrids. Finally, we identified a series of tomato genotypes with different fruit colors, including red, yellow, brown, pink, light-yellow, pink-brown, yellow-green, and light green. We demonstrated that our strategy is efficient and is able to obtain transgene-free plants with different colored fruits in less than 1 year. Most importantly, it does not alter other important agronomic traits, like yield and fruit quality. Our strategy can also be used for manipulating other multigene-controlled traits of tomato and serves as a good example for improving other horticultural crops with multiplex gene editing.

## Results

### Generation of green-fruited *psy1 myb12 sgr1* triple mutant using CRISPR/Cas9

**Figure 2:**
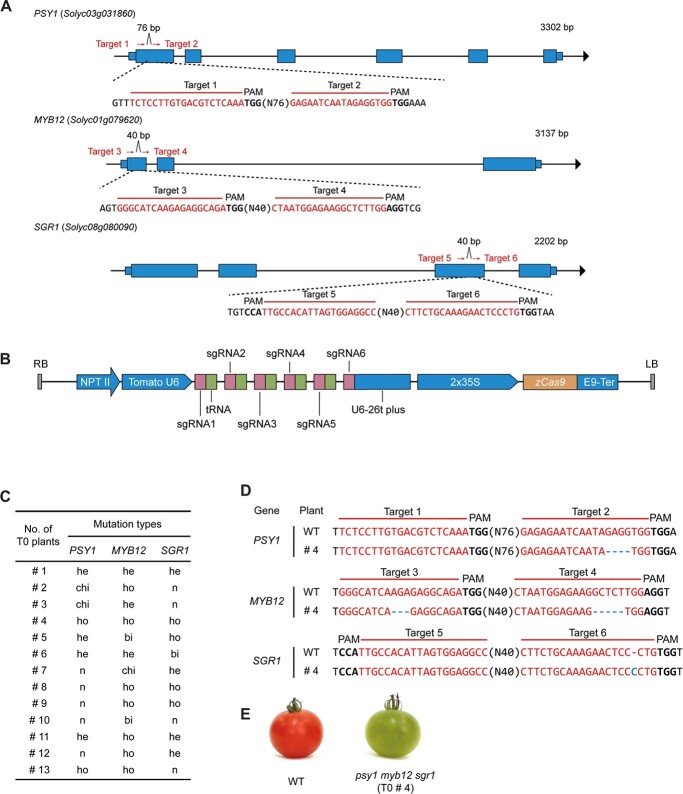
Generation of the *psy1 sgr1 myb12* triple mutant using the CRISPR/Cas9 technology. (A) Schematic showing the exonic regions of tomato fruit color-related genes, *PSY1* (targets 1 and 2), *MYB12* (targets 3 and 4), and *SGR1* (targets 5 and 6), targeted by CRISPR/Cas9. The blue region represents the gene exon. Two target regions separated by 76 and 40 bp were selected in the first exon of *PSY1* and *MYB12* genes, respectively. Similarly, two target regions separated by 40 bp were selected in the third exon of *SGR1*. Letters in red represent the nucleotide sequence of the targeted regions, and letters in bold font indicate the protospacer-adjacent motif (PAM) sequence. (B) Schematic diagram of the vector harboring six sgRNAs in series. *NPTII* served as the resistance marker gene. All six sgRNAs and their corresponding tRNAs were driven by the tomato U6 promoter and terminated by the U6-26t terminator. *zCas9* was driven by the 2 × 35S promoter and terminated by E9-Ter. (C) Summary of 13 T_0_ lines generated using the CRISPR/Cas9 gene-editing system. he, heterozygous; ho, homozygous; n, no mutation; chi, chimeric; bi, biallelic. (D) Comparison of the DNA sequence of T_0_ line #4 triple mutant (*psy1 myb12 sgr1*) with that of the WT. (E) Representative phenotypes of T_0_ line #4 triple mutant (*psy1 myb12 sgr1*) and WT plants.

Given that a novel color can be generated by mixing two or more colors, we hypothesized that mutating multiple fruit color-regulating genes (*PSY1*, *MYB12*, and *SGR1*) in a single genetic background will result in the production of rare-colored fruit. To test this hypothesis and to create new fruit colors in an excellent genetic background, we employed a modified multiplex gene-editing system to edit *PSY1*, *MYB12*, and *SGR1* simultaneously. Taking into consideration that the efficiency of CRISPR/Cas9-based editing varies with the target sequence, two target sites were selected in the exon region of each gene (targets 1 and 2 for *PSY1*, targets 3 and 4 for *MYB12*, and targets 5 and 6 for *SGR1*) (Fig. 2A). A total of six single-guide RNAs (sgRNAs) were cloned together into the pTX041 vector using the Golden Gate assembly method (Fig. 2B), as described in our previous studies [13, 14], and the insertion of sgRNAs was confirmed by sequencing. The resulting construct was transformed into the red-fruited cultivar ‘Ailsa Craig’ through *Agrobacterium*-mediated transformation.

After the recovery of transgenic plants, 13 T_0_ plants (#1–13) were analyzed to detect mutations in the target regions (Fig. 2C). The mutation rate varied widely (7.69–92.30%) among the target regions, and was the highest at target 4 (Supplementary Data Table S1). These results indicate that our CRISPR/Cas9-mediated multiplex gene editing system is extremely efficient in creating custom modifications at the target regions. Additionally, the percentages of T_0_ plants carrying mutations in the three targeted genes were similar to the expected mutation rates (Fig. 2C, Supplementary Data Table S1). This indicates that mutations induced in the three genes by a single construct likely occurred independently of each other and that the levels of Cas9 and sgRNAs were not limiting in the edited tomato plants. When the CRISPR/Cas9 components began to function in the cell after being inserted into the tomato genome, one or both copies of the target genes may have been cleaved and mutated.

Next, to investigate the editing events in T_0_ plants, we examined the putative genotypes using leaf samples. The results showed that homozygous and heterozygous mutations were the most common at all six target sites (Supplementary Data Table S1). As expected, the homozygous triple mutant line #4 (T_0_) harbored mutations in all three genes simultaneously relative to other lines (Fig. 2D). To further examine the specificity of sgRNAs, the potential off-target sites analyzed by Cas-OFFinder [21] were PCR-amplified and sequenced in line #4 (T_0_). No off-target mutations were identified (Supplementary Data Table S2), demonstrating the high specificity of the CRISPR/Cas9 system used in this study.

Finally, we performed phenotypic analysis of tomato fruits. The fruits of line #4 (T_0_), which harbored mutations in all three genes simultaneously, were light green in color (Fig. 2E). This finding indicates that (i) tomato fruit color is regulated by the expression of *PSY1*, *MYB12*, and *SGR1*, which could potentially be used in tomato breeding; and (ii) the combination of *psy1*, *myb12*, and *sgr1* mutant alleles can result in the formation of rare-colored fruits.

### Isolation and identification of tomato genotypes with different fruit colors in the BC_1_F_2_ segregating population

**Figure 3:**
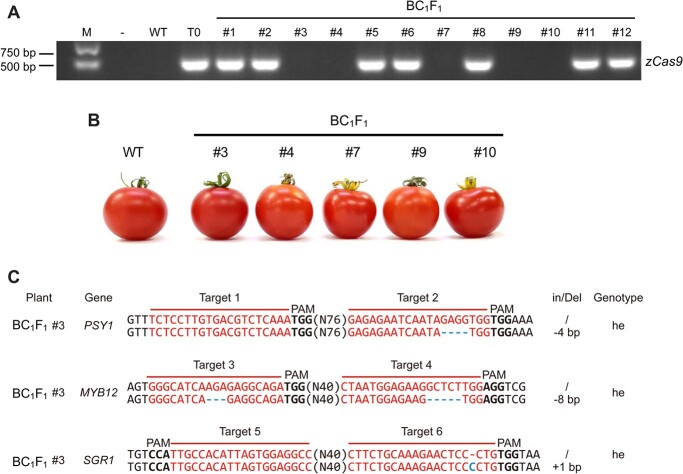
Identification of *zCas9*-lacking heterozygous mutants in the BC_1_F_1_ generation. (A) Exogenously inserted *zCas9* fragments were identified by PCR. The ‘**–**’ symbol indicates that no DNA was added to the PCR reaction. WT and T_0_ plants were used as negative and positive controls, respectively. Line #3, 4, 7, 9, and 10 plants lacked the exogenous *zCas9* fragment. (B) Representative phenotype of WT and BC_1_F_1_ fruits lacking the *zCas9* fragments. Fruits of all BC_1_F_1_ plants were red in color. (C) Sequence analysis of three edited genes (*PSY1*, *MYB12*, and *SGR1*) in BC_1_F_1_ line #3. Mutations in all three genes were heterozygous.

Because z*Cas9* could continue to function in edited plants and produce chimeric DNA, we intended to isolate the *zCas9* gene and other foreign fragments as early as possible. Furthermore, we sought to obtain genotypes homozygous for various combinations of *psy1*, *myb12*, and *sgr1* mutant alleles as quickly as possible. Therefore, we employed a backcross–screening–selfing–segregation strategy, whereby we crossed the green-fruited T_0_ #4 triple mutant with the red-fruited WT plants to generate BC_1_F_1_ hybrids. The presence of *zCas9* was confirmed in 12 randomly selected BC_1_F_1_ individuals by PCR using the primers listed in Supplementary Data Table S3. A 409-bp fragment was amplified from *zCas9*-positive seedlings (T_0_), whereas no amplification was detected in *zCas9*-negative and non-transgenic control (WT) seedlings (Fig. 3A). Like WT plants, five BC_1_F_1_ individuals (BC_1_F_1_ #3, #4, #7, #9, and #10) negative for *zCas9* produced fruits that were red at the ripening stage. These results indicate that all of these five BC_1_F_1_ individuals were heterozygous for the mutation in *PSY1*, *MYB12*, and *SGR1* (Fig. 3B). To verify this result, the DNA of all five BC_1_F_1_ individuals was analyzed by sequencing. As expected, the individuals were heterozygous for mutations in *PSY1*, *MYB12*, and *SGR1*. The gene mutations found in these BC_1_F_1_ individuals were consistent with those found in the T_0_ #4 triple mutant. Additionally, the growth, flowering time, and ripening time of these five BC_1_F_1_ individuals were similar to those of WT plants. Among the five BC_1_F_1_ individuals, BC_1_F_1_ #3 was selected for breeding (Fig. 3C, Supplementary Data Fig. S1).

### Mutations in *PSY1*, *MYB12,* and *SGR1* alter tomato fruit color

**Figure 4:**
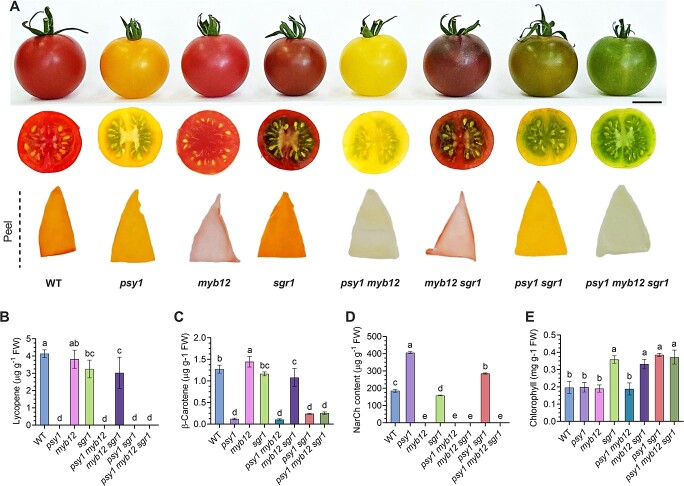
Phenotypic and biochemical analyses of the tomato fruits of WT plants and single- (*psy1*, *myb12*, *sgr1*), double- (*psy1 myb12*, *myb12 sgr1*, *psy1 sgr1*), and triple- (*psy1 myb12 sgr1*) mutant lines in the BC_1_F_2_ generation. (A) Range of the color of tomato fruits obtained in this study. Photographs show the fully mature whole fruits as well as their cross-sections and peel. (B–E) Contents of β-carotene (B), lycopene (C), NarCh (D), and chlorophyll (E) in the fruits of seven isolated homozygous mutant genotypes and the WT. All compounds were quantified at 14 days after the breaker stage (Br + 14). Data represent mean ± standard deviation of three biological replicates. Lower-case letters indicate significant differences (*P* < .05; Student’s *t*-test). Scale bar = 2 cm.

To segregate the *psy1*, *sgr1*, and *myb12* mutant alleles, a segregating BC_1_F_2_ population comprising a total of 288 individuals was developed by selfing BC_1_F_1_ line #3 in a greenhouse. Homozygous single-mutant (*psy1*, *myb12*, and *sgr1*), double-mutant (*psy1 myb12*, *psy1 sgr1*, and *myb12 sgr1*), and triple-mutant (*psy1 myb12 sgr1*) seedlings were identified by PCR with subsequent sequencing (Supplementary Data Table S4). Then, the homozygous seedlings were transplanted to the field, while the heterozygous seedlings were discarded. Because *zCas9* was lost during segregation in the BC_1_F_1_ generation, none of these mutant lines contained the *zCas9* fragment (Supplementary Data Fig. S2). Next, we performed phenotypic analysis of the fruits of these BC_1_F_2_ tomato plants at 14 days after the breaker stage (Br + 14). The results showed that the BC_1_F_2_ plants produced fruits in eight different colors including red, yellow, brown, pink, light-yellow, pink-brown, yellow-green, and light green (Fig. 4A). Genotype–phenotype association analysis revealed that the *psy1*, *myb12*, and *sgr1* single mutants produced yellow, pink, and brown fruits, respectively, consistent with previous studies [10, 13, 22]. However, combination of mutations in any two of the three genes altered the fruit color. For instance, the *psy1 myb12* double mutant produced light-yellow fruits; the *myb12 sgr1* line produced pink-brown fruits; and the *psy1 sgr1* mutant produced yellow-green tomato fruits. Moreover, as we describe in Fig. 2E, the *psy1 myb12 sgr1* triple mutant bore light green fruit.

Thus, different colored fruits were produced by mutant plants. This difference in fruit color among the mutant lines was presumably caused by differences in the level of carotenoid biosynthesis and chlorophyll degradation, which are regulated by *PSY1*, *MYB12*, and *SGR1*. To test this hypothesis, the fruits of different mutant lines were harvested at the Br + 14 stage, and their lycopene and β-carotene contents were determined by high-performance liquid chromatography (HPLC). The results showed that mutants harboring the *psy1* mutant allele contained significantly lower amounts of lycopene and therefore produced yellow or green fruits (Fig. 4A and B). Interestingly, the lycopene content of *sgr1* single-mutant and *sgr1 myb12* double-mutant fruits was significantly lower than that of WT fruits (Fig. 4B), suggesting that *SGR1* is involved in lycopene biosynthesis and metabolism. No significant difference was detected between the lycopene contents of *myb12* single-mutant and WT fruits, demonstrating that *MYB12* is not involved in lycopene synthesis (Fig. 4B).

The red-colored tomato fruit mainly contains lycopene (red carotenoid), which accounts for 90% of the total carotenoid content, and β-carotene (yellow carotenoid). Lycopene is catalyzed by lycopene β-cyclase (CYC-B) to form β-carotene, and the ratio of these two carotenoids affects the fruit color [23]. As expected, the carotenoid content of the *psy1* single mutant was significantly lower than that of other genotypes because of the abnormal accumulation of lycopene (Fig. 4B). Additionally, the mutation in *PSY1* decreased the ratio of lycopene content to carotenoid content, resulting in the fruit appearing yellow (Fig. 4A–C).

Fruit color is the sum of peel and flesh color. The red color of tomato fruit is significantly influenced by the accumulation of the yellow-colored NarCh in the peel and red-colored lycopene in the flesh, which is positively regulated by the *MYB12* gene [10]. In the current study, we found that plants harboring a mutation in *MYB12* accumulated relatively lower amounts of NarCh content in the peel, resulting in lighter-colored fruit (Fig. 4A). Interestingly, we noticed that the peel-specific NarCh content of *sgr1* single-mutant fruit was significantly lower than that of WT fruit (Fig. 4D), suggesting that *MYB12* and *SGR1* play a critical role and positively control the accumulation of NarCh in tomato fruit peel. Our results also showed that the *psy1* single mutant accumulated significantly higher amounts of NarCh in the fruit compared with the WT (Fig. 4D). Similarly, the NarCh content of *psy1 sgr1* double-mutant fruit was significantly higher than that of *sgr1* single-mutant fruit (Fig. 4D). These results indicate that *PSY1* negatively regulates NarCh biosynthesis in the tomato fruit peel.

The process of tomato fruit ripening is accompanied by the accumulation of carotenoids (including lycopene and β-carotene) and the degradation of chlorophyll. Previously, Luo *et al*. [24] confirmed that *SGR1* positively regulates chlorophyll degradation. Consistently, we found that genotypes harboring a mutation in *SGR1* possessed considerably higher chlorophyll content in tissues than other lines, making them lush green (Fig. 4A). However, lines lacking the *sgr1* mutant allele showed no significant difference in chlorophyll content compared with the WT (Fig. 4E), while other mutants harboring *psy1* and *myb12* did not show a significant change in chlorophyll content.

### Effect of *PSY1*, *MYB12,* and *SGR1* on tomato fruit quality and yield

**Figure 5:**
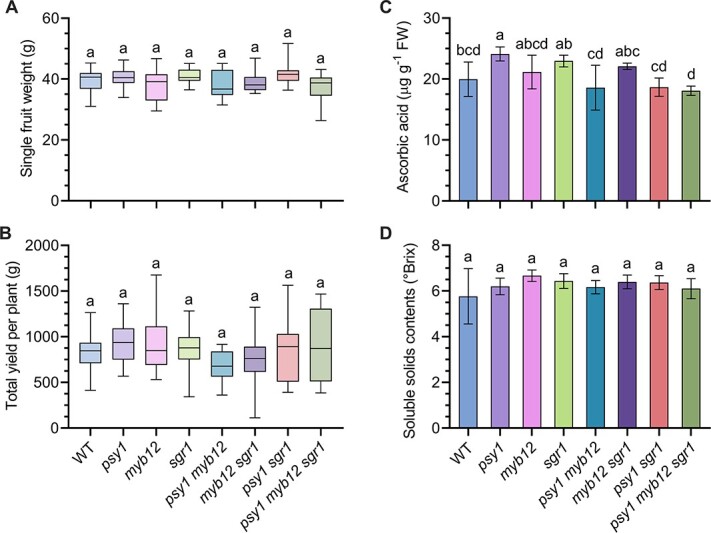
Quantitative and qualitative analyses of the tomato fruits of WT plants and single (*psy1*, *myb12*, *sgr1*), double (*psy1 myb12*, *myb12 sgr1*, *psy1 sgr1*), and triple (*psy1 myb12 sgr1*) mutants in the BC_1_F_1_ generation. (A) Single-fruit weight. (B) Total yield per plant. (C) Ascorbic acid content, expressed as °Brix. (D) TSS content. Measurements were recorded using seven isolated homozygous mutant genotypes and WT plants. Data represent mean ± standard deviation of three biological replicates. Lower-case letters indicate significant differences (*P* < .05; Student’s *t*-test).

Despite the fact that the growth, flowering time, and ripening time of mutant plants remained similar to those of WT plants throughout the growing season, we were concerned that knockout mutation of fruit color-related genes (*PSY1*, *MYB12*, and *SGR1*) may negatively affect other critical agronomic traits including fruit quality and yield. To rule out this concern, the single-fruit weight of each mutant was measured at the Br + 14 stage. The single-fruit weight of each mutant showed no significant difference from that of the WT (Fig. 5A), and fruit yield measurements produced similar results (Fig. 5B). These results suggest that mutations in *PSY1*, *MYB12*, and *SGR1* do not affect tomato yield.

The nutritional value of tomato fruits directly affects their commercial significance. No remarkable variation was detected in the ascorbic acid content of fruits between the WT and mutant lines, except for the *psy1* single mutant, which showed significantly higher ascorbic acid content of fruit compared with other mutants and the WT (Fig. 5C). Furthermore, the total soluble solids (TSS) content of fruits, which is an important indicator of fruit quality, showed no substantial difference in the mutant lines and the WT (Fig. 5D). Taken together, the qualitative data suggest that mutations in *PSY1*, *MYB12*, and *SGR1* do not affect important agronomic traits of the tomato fruit.

## Discussion

Fruit color is one of the most important traits in tomato breeding because customers tend to prefer new fruit colors rather than regular red-colored tomatoes [25]. The color of tomato fruit is influenced mostly by the color of its peel and flesh [10, 25]. Traditional breeding techniques have been successful in integrating diverse fruit color traits into elite tomato varieties through multi-generational backcrossing. However, the cultivation of varieties with different colored fruits requires the aggregation of multiple genetic loci into a single genetic background, making it difficult for breeders to avoid linkage drag and its adverse traits [19]. The introgression process is often slow and labor-consuming because of numerous hurdles, such as genetic barriers and the need for manual emasculation and pollination of flowers, which significantly increase the cost of labor and the duration of the breeding program [19]. Thus, modern breeding strategies are needed for the development of a series of different colored tomato fruits of high commercial value in the background of elite varieties. As a rapid and precise breeding technique, the CRISPR/Cas9-mediated gene-editing system has demonstrated its power in creating complex and polygenic traits underpinned by multiple quantitative loci [26].

In the current study, we developed a breeding strategy (Fig. 1) to accelerate the breeding of diverse tomato fruit colors using the multiplex CRISPR/Cas9 genome editing system to combine six efficiently edited targets in ‘Ailsa Craig’ (Fig. 2). First, we generated the green-fruited triple mutant (*psy1 myb12 sgr1*) from the WT red-fruited ‘Ailsa Craig’ using the CRISPR/Cas9 system. Next, we crossed the green-fruited *psy1 myb12 sgr1* triple mutant (T_0_) to generate BC_1_F_1_ hybrids (Supplementary Data Fig. S1). This allowed us to remove the *zCas9* gene and other foreign DNA fragments from the tomato genome in the shortest time (Fig. 3A). Furthermore, the screening of exogenously inserted Cas9-sgRNA was essential, as its presence could lead to continued gene editing and chimerism. Finally, we self-pollinated the BC_1_F_1_ plants to obtain a segregating BC_1_F_2_ population comprising lines showing stably inherited fruit color (Fig. 4A). Using this breeding strategy, we rapidly created new tomato varieties with the high-quality traits of the genetic background but different fruit colors, which can satisfy the color preference of consumers to a large extent. Notably, compared with the typical advanced backcross breeding method, this approach is more efficient and produced transgene-free variably colored tomato lines in less than 1 year. Most importantly, our approach retains the advantages of elite cultivars and does not cause linkage drag.

Previous studies showed that *PSY1*, *MYB12*, and *SGR1* play a substantial role in the formation of different fruit colors and their pigments [5, 10, 13, 24]. PSY1 is commonly considered to be the rate-limiting enzyme in the biosynthesis of fruit carotenoids [24]. Repression of *PSY1* by antisense silencing in transgenic lines resulted in yellow-colored fruits at the maturity stage, with only 3% of the total carotenoid content of that in the WT fruit [27]. In addition, evidence shows that elevated and constitutive expression of the *PSY1* gene increases the amount of β*-*carotene in WT tomato fruits [28]. Consistently, our results showed that *psy1* mutant fruits contained markedly lower amounts of lycopene and β*-*carotene than other mutant fruits (Fig. 4B and C), and the reduction in these two carotenoids in *psy1* fruit caused it to appear yellow (Fig. 4A). This is consistent with a previous study, which indicated that lycopene accumulation in tomato fruits is absolutely regulated by *PSY1* [16]. Because *PSY1* occupies a critical position in the carotenoid metabolic pathway, its role in other metabolic pathways has been overlooked. In this study, we found that the *psy1* single mutant accumulates more of the yellow-colored flavonoid NarCh than the WT, and a similar trend was observed in the *psy1 sgr1* double mutant relative to the *sgr1* single mutant (Fig. 4D). Consistent with this finding, the fruit of *psy1* showed yellow-colored peel (Fig. 4A). These findings suggest that *PSY1* positively regulates the accumulation of β-carotenoid and lycopene, but negatively regulates the biosynthesis of NarCh. Nevertheless, further investigation is needed to explore the underlying mechanism.

The quality traits of tomato fruit, including taste and nutritional value, directly affect its commercial value. Tomato fruit is an excellent source of ascorbic acid, a critical nutrient that helps maintain the immune system and cannot be synthesized by the human body [29]. Therefore, keeping in mind the importance of ascorbic acid content, the fruits of each genotype were harvested at the Br + 14 stage for quantification. No marked differences in the ascorbic acid content were detected among all mutants (Fig. 5C). Furthermore, the TSS content, which comprises all water-soluble compounds, including monosaccharides, disaccharides, polysaccharides, vitamins, and minerals, is an important indicator of fruit quality. No significant difference was detected in the TSS content (°Brix) between the mutant and WT fruits (Fig. 5D). Agronomic traits, including single-fruit weight and total yield, also showed no marked changes at the Br + 14 stage (Fig. 5A and B). This is consistent with the study of Faria *et al*. [30], which showed that the color development mutants of tomato, old gold-crimson (*ogc*), and high pigment (*hp*), had no effect on total fruit yield or mean fruit mass per plant. Collectively, these findings demonstrate that mutations in *PSY1*, *MYB12*, and *SGR1* do not affect the yield and quality of tomato fruit.

Previously, we obtained purple-fruited tomatoes by overexpressing the *SlAN2-like^InR^* gene [31]. Besides regulating the genes involved in carotenoid, flavonoid, and chlorophyll biosynthesis pathways, the introduction of other pigment biosynthesis genes can also alter tomato fruit color. Polturak *et al*. [32] transferred the betalain synthesis-related genes *CYP76AD1*, *BvDODA1*, and *cDOPA5GT* into tomato plants, which eventually created purple-red-colored tomato fruit. However, random insertion of DNA fragments into the plant genome can lead to unintended effects through the disruption, activation, modification, or silencing of the expression of some endogenous genes [33]. The CRISPR/Cas9 technology enables the accurate and targeted knock-in mutation of the exogenous genes, thus simplifying the genetic background and increasing the precision and efficiency of the experiment. Hence, the use of CRISPR/Cas9-mediated knock-in technology can help create rare fruit colors in the future, which will greatly increase the commercial value of tomatoes [34]. Moreover, the *zCas9* gene, which is considered to be exogenous DNA, needs to be removed from the edited plants by screening, although this process is time-consuming and laborious. A previous study showed that plant genomes can be edited by delivering *in vitro* transcripts or ribonucleoprotein complexes of CRISPR/Cas9, instead of foreign DNA, via particle bombardment [35]. Thus, by combining our CRISPR/Cas9-mediated multiplex genome editing system with a DNA-free genome editing method, we can further shorten the breeding time and improve breeding efficiency.

In conclusion, we propose a breeding strategy to rapidly generate different colored tomato fruits from WT (red-colored) tomato fruit in less than 1 year, thus shortening the duration of breeding, which can take several years. The green-fruited tomato line, created through simultaneous knockout mutations of multiple genes (*PSY1*, *MYB12*, and *SGR1*) in the WT tomato plant using the CRISPR/Cas9 system, was backcrossed with WT plants to generate BC_1_F_1_ hybrids. The obtained progeny were self-pollinated to generate the BC_1_F_2_ population. Knockout mutations of the three above-mentioned genes mainly affected the biosynthesis/accumulation of fruit pigments, without altering other important agronomic traits, such as fruit yield and quality. This strategy can be applied to other multigene-controlled phenotypes and shows great practical potential in breeding and production. This study can be used as a reference for breeding other horticultural crops via multiplex gene editing.

## Materials and methods

### Plant materials and growth conditions

Tomato (*S. lycopersicum*) cultivar ‘Ailsa Craig’ was used as WT in this study. *Agrobacterium tumefaciens* strain LBA4404 was used to transform the cotyledon explants, as described previously [36]. Plants grown in the greenhouse were managed using the same practices as those employed in field production. In the field experiment, tomato plants were topped at the time of four panicles and retained five fruits per panicle.

### Vector construction

To construct the CRISPR/Cas9 binary vector, tRNA was used to space the six sgRNAs and synthesized as described in the previous study [37]. Six sgRNAs targeting the coding sequence of the three genes of interest were designed using the CRISPR-P tool (http://cbi.hzau.edu.cn/cgi-bin/CRISPR). The synthesized sgRNAs were purified and cloned into the pTX041 vector at *Bsa*I sites using the Golden Gate assembly method [38, 39]. The resulting construct was confirmed by sequencing and then introduced into the red-fruited WT plants through *Agrobacterium*-mediated transformation. Primers used for PCR and sequencing are listed in Supplementary Data Table S3.

### Genotyping of transgenic plants

Genomic DNA was extracted from 7-day-old seedling cotyledons using the DNA rapid extraction kit (Biomed, ZF 0100012). Primers (Supplementary Data Table S3) flanking both sgRNA targets were designed for PCR-based genotyping analysis. For T_0_ plants, the PCR amplicon derived from each line was cloned into the pMD18-T vector, and 15 individual clones were sequenced. For BC_1_F_2_ plants, a PCR amplicon derived from each line was directly sequenced. The presence of the T-DNA insert was detected by PCR amplification using a *zCas9*-specific primer pair (Supplementary Data Table S3). Flowers were marked at the peak flowering stage for subsequent fruit quality analysis. The weight of 10 single fruits was measured at the Br + 14 stage.

### Analysis of off-target mutations

Potential off-target sites were predicted using the Cas-OFFinder online tool (Supplementary Data Table S2). A sequence-specific primer pair (Supplementary Data Table S3) was designed for each potential off-target site. PCR products amplified from WT and T_0_ plants were sequenced.

### Determination of pigment contents and nutrition index

To quantify the color-inducing pigments, 10 tomato fruits were harvested at the Br + 14 stage from each plant grown in the greenhouse. Fruit pericarp and peel were peeled off using a pair of tweezers, ground in liquid nitrogen, and then stored at −80°C. Approximately 5 g of the frozen powder was used for liquid chromatography–mass spectrometry (LC–MS) analysis. Ascorbic acid, NarCh, and carotenoid contents were measured as described previously [13]. Chlorophyll a and chlorophyll b contents were measured by HPLC, as described by Shi *et al*. [40]. Individual substances were quantified by comparison with the peak areas of standard substances, and all experiments were performed with three independent biological replicates.

### Determination of total soluble solids content

The TSS content of tomato fruits was measured as described previously [14]. Briefly, 10 tomato fruits were harvested at the Br + 14 stage. The TSS content of the juice of each sample was measured using the Pocket Brix-Acidity Meter (Tomato) (PAL-BX/ACID3, Atago), and recorded in °Brix.

## Supplementary Material

Web_Material_uhac214

## Data Availability

All datasets generated in this study are included in the article and supplementary materials.
