# Supplementary material for: Recoloring tomato fruit by CRISPR/Cas9-mediated multiplex gene editing
Source: Hortic Res. 2022 Sep 19;10(1):uhac214. doi: 10.1093/hr/uhac214 (PMC9832834; doi:10.1093/hr/uhac214)
Supplement: Web_Material_uhac214 [file web_material_uhac214.zip › Supplementary Figures.docx]

**Recoloring tomato fruit by CRISPR/Cas9-mediated multiplex gene editing**

Tianxia Yang^1,2,†^, Muhammad Ali^1,2,†^, Lihao Lin^1,2,†^, Ping Li^3^, Hongju He^4^, Qiang Zhu^1,2^, Chuanlong Sun^1,2^, Ning Wu^1,2^, Xiaofei Zhang^1,2^, Tingting Huang^3^, Chang-Bao Li^5^, Chuanyou Li^1,2,*^, Lei Deng^1,2,*^

^1^State Key Laboratory of Plant Genomics, National Center for Plant Gene Research (Beijing), Institute of Genetics and Developmental Biology, Innovation Academy for Seed Design, Chinese Academy of Sciences, Beijing 100101, China

^2^CAS Center for Excellence in Biotic Interactions, University of Chinese Academy of Sciences, Beijing 100049, China

^3^Institute of Vegetable, Qingdao Academy of Agricultural Sciences, Qingdao, Shandong Province 266100, China

^4^Institute of Agri-food Processing and Nutrition, Beijing Academy of Agriculture and Forestry Sciences, Beijing 100097, China

^5^Key Laboratory of Biology and Genetic Improvement of Horticultural Crops (North China), Ministry of Agriculture, Beijing Vegetable Research Center, Beijing Academy of Agriculture and Forestry Sciences, Beijing 100097, China

^†^These authors contributed equally to this work.

^*^Corresponding authors: Chuanyou Li ([cyli@genetics.ac.cn](mailto:cyli@genetics.ac.cn)), Lei Deng ([ldeng@genetics.ac.cn](mailto:ldeng@genetics.ac.cn))

**Supplementary Figure S1.** Fruit phenotypes of the wild type (WT), *psy1 myb12 sgr1* triple mutant (line#4), and their BC_1_F_1_ and BC_1_F_2_ progeny.


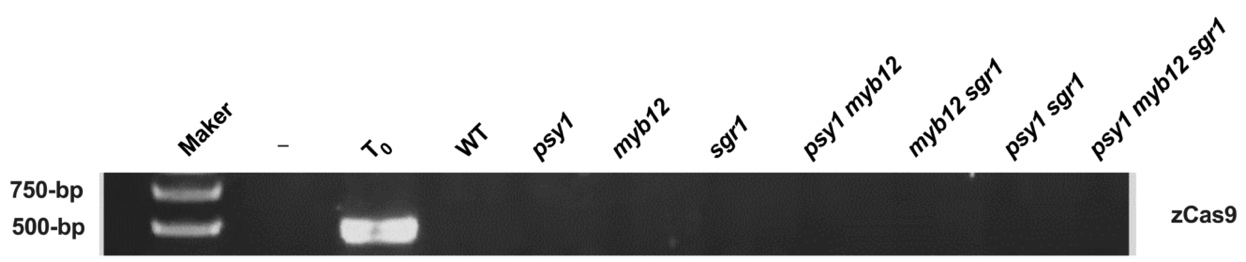


**Supplementary Figure S2.** Detection of the z*Cas9* fragment in T_0_, WT, and BC_1_F_2_ mutant lines. No *zCas9* residue was found in the WT and mutant lines.
